# Body Image in Adolescents with Gender Incongruence and Its Association with Psychological Functioning

**DOI:** 10.3390/ijerph20043349

**Published:** 2023-02-14

**Authors:** Anouk Verveen, Anna I. R. van der Miesen, Nastasja M. de Graaf, Baudewijntje P. C. Kreukels, Annelou L. C. de Vries, Thomas D. Steensma

**Affiliations:** 1 Centre of Expertise on Gender Dysphoria, Amsterdam UMC Location Vrije Universiteit Amsterdam, De Boelelaan 1117, 1081 HZ Amsterdam, The Netherlands; 2 Department of Medical Psychology, Amsterdam UMC Location Vrije Universiteit Amsterdam, De Boelelaan 1117, 1081 HZ Amsterdam, The Netherlands; 3 Department of Child and Adolescent Psychiatry, Amsterdam UMC Location Vrije Universiteit Amsterdam, De Boelelaan 1117, 1081 HZ Amsterdam, The Netherlands

**Keywords:** adolescents, gender incongruence, body image, body satisfaction, psychological functioning

## Abstract

During adolescence, many individuals with gender incongruence (GI) experience distress related to body dissatisfaction. This study aims to describe the body (dis)satisfaction of Dutch adolescents referred for GI and to describe the influence of body image on their psychological functioning. Self-report measures on body satisfaction (Body Image Scale) and psychological functioning (Youth Self-Report) were obtained from 787 adolescents (aged 10–18) who were referred to the Center of Expertise on Gender Dysphoria at the Amsterdam University Medical Centers between 1996 and 2016. First, a general description of body satisfaction in adolescents with GI was developed. Secondly, multiple linear regression analyses were performed to determine the association between body image and psychological functioning, both for total problems and for internalizing and externalizing problems separately. Third, regression analyses are repeated for body area subscales. Adolescents with GI report the greatest dissatisfaction with the genital area, regardless of birth-assigned sex. For all other body areas, there were birth-assigned sex differences in satisfaction. The analyses showed that body satisfaction was significantly related to total psychological problems and both internalizing and externalizing problems. Greater body dissatisfaction is significantly associated with worse psychological functioning in adolescents with GI. Clinicians should monitor the body image of adolescents with GI over time, especially during puberty and medical interventions.

## 1. Introduction

Usually, sex is identified by sex chromosomes, gonads, and genitalia [1]. Gender identity is defined as the experience of being or belonging to a gender. For some individuals, their gender identity is not congruent with their birth-assigned sex, and they experience gender incongruence (GI) [2]. Gender may play a role in many different aspects in life, for example how young people experience their identity, their interaction with their peers, as well as how they view themselves, especially throughout adolescence [3,4]. Around puberty, many adolescents with GI experience distress related to body dissatisfaction, which may have an impact on body image [5].

Body image can be defined as a person’s self-concept, consisting of attitudes, experiences, and perceptions pertaining to one’s physical appearance, in relation to the social context [6]. Previous research has shown that adults with GI were more likely to evaluate their body more negatively, have higher levels of body insecurity, and higher levels of sexual-physical discomfort compared to the general population [7,8]. More specifically, adults with GI experience more dissatisfaction with gender-specific socially related body parts (such as voice), and sex-related body parts (e.g., primary and secondary sex characteristics), than with their neutral body parts (such as feet and nose) [9,10]. In addition, adults with GI were also more dissatisfied with non-sex-specific characteristics and overall appearance compared to the general population [10,11].

When looking at sex assigned at birth differences in adults with GI, it has been observed that individuals assigned male at birth (AMAB) reported more body dissatisfaction than birth-assigned females (AFAB) [8,10]. The areas of discomfort also differed between the two groups. Those AMAB were most dissatisfied with their socially related body parts, such as voice and posture, and those AFAB were most dissatisfied with their breasts, hip, and chest region [10,12]. For adolescents experiencing GI, a previous study found that individuals AFAB were more dissatisfied with their primary and secondary sex characteristics than individuals AMAB [13].

As described in the literature, experiences of body image and psychological functioning are related. In studies on adolescents from the general population, body dissatisfaction was found to be a significant risk factor for having psychological problems, such as eating disorders and depression [14,15,16]. Similarly, in adults with GI, a negative body image was related to lower self-esteem and poorer social functioning [10,17]. Body dissatisfaction increases the risk of developing eating disorders in adults with GI [12,16].

In addition, it is well known that adolescents with GI tend to experience emotional and behavioral problems [3]. In clinic-referred samples, internalizing problems, such as mood and anxiety disorders, were more common than externalizing problems, such as rule-breaking or aggressive behavior [3]. In these adolescent samples, internalizing problems were generally more prevalent in those AMAB than those AFAB, who showed more externalizing problems [3]. Furthermore, it is also established that adolescents with GI experience distress at the time their body starts to develop. For adolescents, the distress from GI becomes more prevalent around the time of puberty, as the incongruence between experienced and desired gender becomes more present [18,19], which has an impact on the growing dissatisfaction around gendered body characteristics [20]. Earlier studies have shown that both greater internalizing problems and lower body satisfaction separately predict lower quality of life in adolescents with GI [10,21]. Therefore, the high levels of body dissatisfaction and psychological problems in adolescents with GI are alarming.

Previous studies have highlighted the importance of assessing the body image of individuals with GI in relation to mental health outcomes [7]. To the best of our knowledge, no studies have been performed on the association between body dissatisfaction and the psychological functioning of adolescents with GI. Therefore, the main research objectives of this study are (1) to describe body (dis)satisfaction of Dutch adolescents referred for GI and determine birth-assigned sex differences; (2) to describe the association between body image and psychological functioning of adolescents with GI; (3) to determine if there is a specific body area that is associated with psychological functioning. It is hypothesized that adolescents who report greater body dissatisfaction also report worse psychological functioning, regardless of the body area.

## 2. Materials and Methods

### 2.1. Participants and Procedure

Between 1996 and 2016, 1188 adolescents were referred to the Center of Expertise on Gender Dysphoria at the Amsterdam University Medical Center (location VUmc), in Amsterdam, The Netherlands. Every individual that is referred to the Center of Expertise starts with an assessment period that includes, in addition to consultations, structural information collection with questionnaires.

All individuals that completed the measures for assessing body image as well as psychological functioning were included in this study. Participants were excluded when more than 20% of the questionnaire items were missing, which led to the exclusion of 401 adolescents. This resulted in a total sample of 787 adolescents between the ages of 10 and 18, of which 297 were AMAB and 490 were AFAB. As all data for this study were collected at intake, the adolescents had not yet received any medical gender-affirming treatment. All data were anonymized and treated confidentially. This study does not fall under the scope of the Dutch Medical Research Involving Human Subjects Act (WMO) and does not require ethical approval. Participants provided informed consent following the good clinical practice guidelines.

### 2.2. Measures

During the adolescent’s period of assessment at the center, the following questionnaires assessing demographics, IQ, gender identity, body image, and psychological functioning were administered:

Demographics and IQ: Sociodemographic variables that were collected include age at intake, birth-assigned sex, parents’ marital status, and total IQ. The parents’ marital status was classified as “two biological parents” and “other” (for example, single parent, step-parent, adopted, or foster care). IQ was assessed using the Dutch versions of the Wechsler Preschool and Primary Scale of Intelligence or the Wechsler Intelligence Scale for Children [22,23].

Body image: The Body Image Scale (BIS) was developed specifically for assessing the body satisfaction of people with GI [24]. In the questionnaire, 30 body characteristics are listed, which the participant rates on a five-point scale of satisfaction from 1 (most satisfied) to 5 (most dissatisfied) [24]. In 2016, van de Grift et al. formulated six scales based on body areas within the BIS [10]. Therefore, the following body area subscales will be used in the current study: (1) social and hair items, (2) head and neck region, (3) muscularity and posture, (4) hip region, (5) chest region, and (6) genitals. The BIS has a good reliability [24]. Mean scores and standard deviations were calculated for the total questionnaire and the six subscales of van de Grift et al. (Cronbach’s alpha 0.72–0.85) [10].

Psychological functioning: The Youth Self-Report (YSR) is a standardized measure of the psychological functioning of young people aged 6–18 years [25,26]. It includes behavioral and emotional problems and is self-reported. It consists of 102 items that are scored on a 0- to 2-point response scale (0 = not true; 1 = sometimes true; and 2 = very true), based on which three scales can be calculated: Internalizing, Externalizing, and Total Problem Scale. Standardized *T*-score values were used. Additionally, clinical range scores were reported for the scales. *T*-score values above 63 were scored in the clinical range [26]. It has been confirmed that the Dutch translation of the YSR has good reliability and validity [26].

### 2.3. Statistical Analyses

Differences between individuals AMAB and individuals AFAB in sociodemographic variables were explored with the use of chi-square tests and *t*-tests. An ANCOVA was used to determine birth-assigned sex differences in BIS total and subscale scores, correcting for the age and marital status of the parents. Zero-order correlations were performed to determine which demographic variables had a significant correlation with the outcome variable and therefore should be included in further analyses.

To answer the research question, multiple linear regression analyses were performed with body image as the independent variable and psychological functioning as the dependent variable to examine whether body image was significantly associated with psychological functioning. YSR mean total problem *T*-scores and internalizing and externalizing *T*-scores were analyzed with the use of a linear regression analysis. Furthermore, linear regression analyses were performed with the six body area subscales of the BIS and the dependent variable YSR total problem *T*-score to determine whether the association differs between body area subscales. For analyses on the subscales, Bonferroni corrections were performed. Finally, logistic regression analyses were performed on the association between body image and YSR clinical range scores for total, internalizing, and externalizing problems.

Statistical analyses were performed with IBM SPSS Statistics version 24. An alpha (α) of 0.05 was used to determine statistical significance.

## 3. Results

### 3.1. Excluded Sample

We checked for differences in demographics between the excluded (*n* = 401) and included (*n* = 787) groups. The mean IQ of the included adolescents was significantly higher (M = 99.93, SD = 15.754) than that of the excluded adolescents (M = 96.94, SD = 16.497), *t*(937) = −2.414, *p* = 0.016. Adolescents from the included group more often lived with both biological parents (59.3%) than adolescents from the excluded group (51.3%), X²(1) = 4.993, *p* = 0.025. There were no significant differences in birth-assigned sex or age at intake.

### 3.2. Sample Characteristics

The demographic characteristics of the study population are presented in Table 1 for the total group and separated for birth-assigned sex. On average, individuals AFAB were older at intake than individuals AMAB (*t*(574) = −3.443, *p* = 0.01). Those AMAB more often lived with both biological parents (χ²(1) = 4.335, *p* = 0.037). Age at intake and marital status of the parents were thus included as covariates in all further analyses.

### 3.3. Body Image of Adolescents with GI

Table 2 shows the means and standard deviations of the measure of body satisfaction, for the total sample and separately for birth-assigned sex. There was no significant difference in the scores for individuals AFAB and AMAB on the mean total score of the Body Image Scale. ANCOVA showed that, whilst adjusting for age and marital status of the parents, there was a significant difference in body satisfaction between AMAB and AFAB adolescents on the scales of social and hair items (F(1, 770) = 43.869, *p* < 0.001), head and neck region (F(1, 771) = 44.185, *p* < 0.001), muscularity and posture (F(1, 771) = 9.010, *p* = 0.003), hip region (F(1, 771) = 78.367, *p* < 0.001), and chest region (F(1, 770) = 72.103, *p* < 0.001). Those AFAB were more dissatisfied with their hip and chest region, while those AMAB expressed more dissatisfaction with social and hair items, head and neck region, and muscularity and posture. There was no birth-assigned sex difference for the subscale genitals.

### 3.4. Body Image and Psychological Functioning

Psychological functioning scores can be found in Table 3. BIS effects on YSR *T*-scores were examined using multiple linear regression analyses. The outcomes are shown in Table 4. Step 1 included the demographic control variables (i.e., birth-assigned sex, age, marital status of the parents, and IQ) as independent variables. Birth-assigned sex, age, marital status of the parents, and IQ were significant in the model. Adolescents had a higher YSR total score when they were AMAB, of older age, had a lower IQ, and when they did not live with both biological parents. For the internalizing score, associations were found with being AMAB, older, and having a lower IQ. Adolescents had a higher externalizing score when they did not live with both biological parents and when they had a lower IQ. Step 2 added the BIS total score as an independent variable and showed a significant association with total, internalizing, and externalizing YSR *T*-score, with a higher score on the BIS being associated with a higher score on the YSR. No interaction effects were found with birth-assigned sex or age at intake.

Separate multiple linear regression analyses were performed in step 1 on birth-assigned sex, age, marital status of the parents, and IQ, and in step 2 on each of the six body area subscales of the BIS. The results are shown in Table S1. All scales but the genitals one were significantly associated with the YSR total *T*-score: social and hair items (F(5, 709) = 17.860, *p* < 0.001), head and neck region (F(5, 710) = 15.220, *p* < 0.001), muscularity and posture (F(5, 710) = 16.539, *p* < 0.001), hip region (F(5, 710) = 16.032, *p* < 0.001), and chest region (F(5, 709) = 13.236, *p* < 0.001). After Bonferroni correction, the effect of the chest region on the YSR total *T*-score was no longer significant. All scales but the genital one were significantly associated with the YSR internalizing *T*-score but only the muscularity and posture subscale was significantly associated with YSR externalizing *T*-score. This effect did not remain significant after a Bonferroni correction, while all relations with the internalizing scale remained. Greater body dissatisfaction on any of the body area scales but the genital one was related to a higher *T*-score on the YSR total scale. No interaction effects with birth-assigned sex or age were found.

Multiple logistic regression analyses with YSR clinical range as the outcome variable were performed. Step 1 included demographic control variables as predictors. Step 2 included the BIS total score as a predictor variable and showed a significant association with the clinical range for YSR total and internalizing problems, where a higher score on the BIS was related to higher odds of being in the clinical range for emotional and behavioral problems. There was no significant association with the externalizing clinical range score. The results are shown in Table 5.

## 4. Discussion

In this study, body image, including birth-assigned sex differences, and the association between psychological functioning of adolescents with GI and their body image were explored.

The first aim of our study was to describe the body (dis)satisfaction of Dutch adolescents referred for GI and determine birth-assigned sex differences. For the total group overall, dissatisfaction with the genital area was much higher than the other body areas, which has been described previously [27]. This has also been found in studies on Dutch children and adults with GI [10,28]. When looking into birth-assigned sex differences, AMABs reported the strongest mean dissatisfaction with genitals, chest region, and “social and hair growth items”. AFABs reported the strongest dissatisfaction with their chest area, hips, and genital body characteristics. Interestingly, the genital subscale was the only body area that did not show birth-assigned sex differences. Adolescents with GI show great dissatisfaction with their genital area, regardless of birth-assigned sex. Evidently, genital anatomy belongs to the primary sex characteristics being present from birth, whereas secondary sex characteristics such as chest, hair growth, and hips are only subject to change when puberty has started.

In line with the second hypothesis of our study, the main finding was that body dissatisfaction was found to be associated with experiencing clinically relevant psychological problems in adolescents with GI. These outcomes are in line with existing literature from the general population, which has shown that body dissatisfaction is associated with psychological problems, such as depressive mood, eating disorders, and low self-esteem [15].

Regarding the third aim of the study, we found an association with total and internalizing psychological problems for all body areas but the genital area. We hypothesize that the absence of an effect for the genital area is due to a ceiling effect, as there was little variability in this scale. For adolescents with GI, it has previously been described that the sex-specific physical changes and anticipated feminization or masculinization that occur during puberty can cause distress [29,30,31]. This distress can lead to psychological problems in this population. This can clearly be seen in the literature on adolescents with GI, which shows us that there is a high vulnerability to experiencing emotional and behavioral problems at enrolment at a gender clinic, related to the development of puberty and body dissatisfaction [3,32]. As expected, this study showed us that greater body dissatisfaction is also significantly associated with worse psychological functioning in adolescents with GI. In a British study on gender clinic-referred adults, the predictive value of negative body image has also been described [17].

Previous studies have shown that adolescents with GI may have a less favorable body image than adults with GI [7] but they seem to have fewer psychological problems than adults with GI [33]. Though body image is an important predictor of psychological functioning, it is not the only one. Other factors such as poor peer relations, stigmatization, and family environment are also predictors of psychological functioning [4,34]. Further studies should take these factors into account when describing body satisfaction and psychological functioning.

A limitation of this study is that the puberty status of the adolescents at intake was unknown. As a result, this could not be adjusted for. Moreover, it is important to remain aware of the heterogeneity of the transgender population and the multiple gender identities in the population, which is known to be related to body satisfaction and psychological functioning [35,36,37].

## 5. Conclusions

In conclusion, high levels of body dissatisfaction can lead to a negative body image and worse psychological functioning in adolescents with GI. Furthermore, it is a significant predictor of lower quality of life [21]. Therefore, it is important to monitor the body image of adolescents with GI over time, especially during puberty and medical interventions as the accompanying physical changes have an effect on body image [31,38]. Treatment with gonadotropin-releasing hormone agonists for puberty suppression can be an important element of treatment to reduce body dissatisfaction and psychological suffering. Apart from medical affirming treatment, psychological guidance aiming at a positive body image can also be beneficial [39].

## Figures and Tables

**Table 1:** Descriptive characteristics of the study population: total sample and separated based on birth-assigned sex.

	Total Sample (*n* = 787)	AMAB (*n* = 297)	AFAB (*n* = 490)
Age at intake (in years) (M, SD) *n* = 787	14.73 (2.11)	14.39 (2.23) **	14.94 (2.01) **
Child lives with (%, *n*) *n* = 776			
Both biological parents	59.3 (460)	64.2 (188) *	56.3 (272) *
Other	40.7 (316)	35.8 (105) *	43.7 (211) *
IQ (M total, SD) *n* = 724	99.93 (15.75)	99.82 (16.02)	99.99 (15.62)

* Significant birth-assigned sex difference at the 0.05 level; ** Significant birth-assigned sex difference at the 0.01 level; AMAB = assigned male at birth, AFAB = assigned female at birth.

**Table 2:** Rating of body image satisfaction: total sample and separated for birth-assigned sex.

	Total Sample (*n* = 787)	AMAB (*n* = 297)	AFAB (*n* = 490)
Body Image Scale (M, SD)			
Total score	3.10 (0.66)	3.07 (0.71)	3.12 (0.62)
Social and hair items	3.05 (0.82)	3.20 (0.90) **	2.95 (0.76) **
Head and neck region	2.59 (0.77)	2.75 (0.85) **	2.49 (0.69) **
Muscularity and posture	2.71 (0.73)	2.73 (0.74) **	2.70 (0.72) **
Hip region	3.27 (1.01)	2.83 (0.94) **	3.53 (0.95) **
Chest region	3.92 (0.96)	3.51 (1.01) **	4.17 (0.83) **
Genitals	4.43 (0.79)	4.43 (0.91)	4.44 (0.71)

** Significant birth-assigned sex difference at the 0.01 level; AMAB = assigned male at birth, AFAB = assigned female at birth.

**Table 3:** Rating of psychological functioning: total sample and separated for birth-assigned sex.

	Total Sample (*n* = 787)	AMAB (*n* = 297)	AFAB (*n* = 490)
Youth Self-Report (YSR) (*T*-score, SD)			
Total score	56.67 (9.94)	57.89 (10.48) **	55.92 (9.53) **
Internalizing	58.08 (11.45)	60.86 (11.51) **	56.39 (11.10) **
Externalizing	50.87 (9.55)	49.85 (9.60) *	51.49 (9.48) *
YSR clinical range (%, *n*)			
Total score	30.1 (237)	36.4 (108) **	26.3 (129) **
Internalizing	35.1 (276)	46.8 (139) **	28.0 (137) **
Externalizing	11.2 (88)	8.8 (26)	12.7 (62)

* Significant birth-assigned sex difference at the 0.05 level; ** Significant birth-assigned sex difference at the 0.01 level; AMAB = assigned male at birth, AFAB = assigned female at birth.

**Table 4:** Multiple linear regression for BIS total scale and YSR *T*-scores.

	**Total YSR** ***T*** **-Score Multivariate Model 1**						
	**B**	**SE**	**Beta**	**t**	**Sig.**	**95% CI B** **Lower Bound**	**95% CI B** **Upper Bound**
(Constant)	53.045	3.687		14.385	0.000	45.805	60.284
Birth-assigned sex	−3.018	0.762	−0.144	−3.962	0.000	−4.514	−1.522
Age at intake	0.942	0.176	0.197	5.365	0.000	0.597	1.287
Marital status	1.640	0.753	0.080	2.177	0.030	0.161	3.118
Total IQ	−0.076	0.023	−0.119	−3.271	0.001	−0.121	−0.030
	**Total YSR** ***T*** **-score Multivariate Model 2**						
	**B**	**SE**	**Beta**	**t**	**Sig.**	**95% CI B** **Lower Bound**	**95% CI B** **Upper Bound**
(Constant)	51.763	3.636		14.236	0.000	44.624	58.901
Birth-assigned sex	−2.844	0.750	−0.136	−3.791	0.000	−4.317	−1.371
Age at intake	0.419	0.202	0.087	2.074	0.038	0.022	0.815
Marital status	1.495	0.741	0.073	2.017	0.044	0.040	2.951
Total IQ	−0.086	0.023	−0.134	−3.758	0.000	−0.131	−0.041
BIS (M)	3.206	0.642	0.209	4.996	0.000	1.946	4.466
	**Internalizing YSR** ***T*** **-score** **Multivariate Model** **1**						
	**B**	**SE**	**Beta**	**t**	**Sig.**	**95% CI B** **Lower Bound**	**95% CI B** **Upper Bound**
(Constant)	50.279	4.148		12.121	0.000	42.135	58.423
Birth-assigned sex	−5.861	0.857	−0.243	−6.839	0.000	−7.544	−4.179
Age at intake	1.423	0.197	0.258	7.206	0.000	1.035	1.811
Marital status	1.311	0.847	0.055	1.547	0.122	−0.352	2.975
Total IQ	−0.054	0.026	−0.073	−2.064	0.039	−0.105	−0.003
	**Internalizing YSR** ***T*** **-score** **Multivariate Model** **2**						
	**B**	**SE**	**Beta**	**t**	**Sig.**	**95% CI B** **Lower Bound**	**95% CI B** **Upper Bound**
(Constant)	48.487	4.051		11.969	0.000	40.534	56.441
Birth-assigned sex	−5.618	0.836	−0.233	−6.722	0.000	−7.259	−3.977
Age at intake	0.692	0.225	0.125	3.076	0.002	0.250	1.134
Marital status	1.109	0.826	0.047	1.343	0.180	−0.513	2.731
Total IQ	−0.068	0.025	−0.092	−2.670	0.008	−0.118	−0.018
BIS (M)	4.481	0.715	0.253	6.268	0.000	3.077	5.884
	**Externalizing YSR** ***T*** **-score** **Multivariate Model** **1**						
	**B**	**SE**	**Beta**	**t**	**Sig.**	**95% CI B** **Lower Bound**	**95% CI B** **Upper Bound**
(Constant)	50.199	3.591		13.978	0.000	43.149	57.250
Birth-assigned sex	1.147	0.742	0.058	1.546	0.122	−0.309	2.604
Age at intake	0.0297	0.171	0.065	1.739	0.082	−0.038	0.633
Marital status	1.610	0.734	0.082	2.194	0.029	0.169	3.050
Total IQ	−0.078	0.023	−0.129	−3.470	0.001	−0.122	−0.034
	**Externalizing YSR** ***T*** **-score Multivariate Model 2**						
	**B**	**SE**	**Beta**	**t**	**Sig.**	**95% CI B** **Lower Bound**	**95% CI B** **Upper Bound**
(Constant)	49.697	3.593		13.832	0.000	42.643	56.751
Birth-assigned sex	1.216	0.741	0.061	1.640	0.101	−0.240	2.671
Age at intake	0.092	0.200	0.020	0.463	0.644	−0.299	0.484
Marital status	1.553	0.733	0.079	2.120	0.034	0.115	2.991
Total IQ	−0.082	0.023	−0.135	−3.640	0.000	−0.127	−0.038
BIS (M)	1.257	0.634	0.086	1.982	0.048	0.012	2.502

Model 1: socio-demographic characteristics; Model 2: socio-demographic characteristics and total BIS score. SE = standard error, 95% CI = 95% confidence interval, BIS = Body Image Scale, YSR = Youth Self-Report.

**Table 5:** Multiple logistic regression for BIS total score and YSR clinical range scores.

	**Clinical Range YSR Total Multivariate Model 1**							
	**B**	**SE**	**Wald**	**df**	**Sig.**	**Exp(B)**	**95% CI Exp(B)** **Lower Bound**	**95% CI Exp(B)** **Upper Bound**
(Constant)	−3.422	0.632	29.312	1	0.000	0.033		
Birth-assigned sex	0.617	0.165	13.990	1	0.000	1.854	1.342	2.563
Age at intake	0.175	0.040	19.143	1	0.000	1.192	1.102	1.289
Marital status	−0.435	0.162	7.190	1	0.007	0.647	0.471	0.890
	**Clinical** **Range YSR Total** **Multivariate Model** **2**							
	**B**	**SE**	**Wald**	**df**	**Sig.**	**Exp(B)**	**95% CI Exp(B)** **Lower Bound**	**95% CI Exp(B)** **Upper Bound**
(Constant)	−3.932	0.662	35.280	1	0.000	0.020		
Birth-assigned sex	0.603	0.166	13.131	1	0.000	1.828	1.319	2.534
Age at intake	0.099	0.046	4.710	1	0.030	1.104	1.010	1.208
Marital status	−0.417	0.163	6.509	1	0.011	0.659	0.479	0.908
BIS (M)	0.519	0.150	11.973	1	0.000	1.680	1.252	2.253
	**Clinical Range YSR Internalizing Multivariate Model 1**							
	**B**	**SE**	**Wald**	**df**	**Sig.**	**Exp(B)**	**95% CI Exp(B)** **Lower Bound**	**95% CI Exp(B)** **Upper Bound**
(Constant)	−4.562	0.640	50.813	1	0.000	0.010		
Birth-assigned sex	1.055	0.165	40.822	1	0.000	2.872	2.078	3.969
Age at intake	0.248	0.040	38.026	1	0.000	1.282	1.185	1.387
Marital status	−0.270	0.161	2.797	1	0.094	0.764	0.557	1.047
	**Clinical Range YSR Internalizing Multivariate Model 2**							
	**B**	**SE**	**Wald**	**df**	**Sig.**	**Exp(B)**	**95% CI Exp(B)** **Lower Bound**	**95% CI Exp(B)** **Upper Bound**
(Constant)	−5.548	0.694	63.945	1	0.000	0.004		
Birth-assigned sex	1.069	0.169	39.781	1	0.000	2.911	2.089	4.058
Age at intake	0.127	0.046	7.719	1	0.005	1.136	1.038	1.242
Marital status	−0.239	0.165	2.116	1	0.146	0.787	0.570	1.087
BIS (M)	0.874	0.157	31.177	1	0.000	2.397	1.763	3.257
	**Clinical Range YSR Externalizing Multivariate Model 1**							
	**B**	**SE**	**Wald**	**df**	**Sig.**	**Exp(B)**	**95% CI Exp(B)** **Lower Bound**	**95% CI Exp(B)** **Upper Bound**
(Constant)	−2.218	0.889	6.221	1	0.013	0.109		
Birth-assigned sex	−0.328	0.250	1.717	1	0.190	0.721	0.441	1.176
Age at intake	0.040	0.057	0.483	1	0.487	1.040	0.930	1.164
Marital status	−0.616	0.232	7.058	1	0.008	0.540	0.343	0.851
	**Clinical Range YSR Externalizing Multivariate Model 2**							
	**B**	**SE**	**Wald**	**df**	**Sig.**	**Exp(B)**	**95% CI Exp(B)** **Lower Bound**	**95% CI Exp(B)** **Upper Bound**
(Constant)	−2.172	0.906	5.754	1	0.016	0.114		
Birth-assigned sex	−0.325	0.250	1.691	1	0.193	0.722	0.442	1.179
Age at intake	0.048	0.065	0.538	1	0.463	1.049	0.923	1.192
Marital status	−0.618	0.232	7.100	1	0.008	0.539	0.342	0.849
BIS (M)	−0.053	0.206	0.066	1	0.797	0.948	0.633	1.421

Model 1: socio-demographic characteristics; Model 2: socio-demographic characteristics and total BIS score. SE = standard error, 95% CI = 95% confidence interval, BIS = Body Image Scale, YSR = Youth Self-Report.

## Data Availability

Anonymized data will be made available upon reasonable request to the corresponding author.

## Supplementary Materials

The following supporting information can be downloaded at: https://www.mdpi.com/article/10.3390/ijerph20043349/s1, Table S1: Multiple linear regression for BIS body area subscales and YSR *T*-scores.
