# Supplementary material for: Body Image in Adolescents with Gender Incongruence and Its Association with Psychological Functioning
Source: Int J Environ Res Public Health. 2023 Feb 14;20(4):3349. doi: 10.3390/ijerph20043349 (PMC9963492; doi:10.3390/ijerph20043349)
Supplement: Supplementary file 1 [file ijerph-20-03349-s001.zip › ijerph-2111293-supplementary.pdf]

SUPPLEMENTARY MATERIALS

Table S1: Multiple linear regression for BIS body area subscales and YSR *T*-scores.

		Total YSR *T*-scores					Internalizing YSR *T*-scores					Externalizing YSR *T*-scores				
		B	SE	Beta	t	Sig.	B	SE	Beta	t	Sig.	B	SE	Beta	t	Sig.
Social and hair items	(Constant)	51.865	3.626		14.304	0.000	48.432	4.000		12.107	0.000	49.854	3.592		13.879	0.000
	Birth-assigned sex	-2.044	0.770	-0.098	-2.653	0.008	-4.328	0850	-0.180	-5.093	0.000	1.397	0.763	0.070	1.831	0.068
	Age at intake	0.415	0.201	0.087	2.066	0.039	0.590	0.221	0.107	2.666	0.008	0.176	0.199	0.039	0.886	0.376
	Marital status	1.734	0.740	0.084	2.344	0.019	1.452	0.816	0.061	1.779	0.076	1.671	0.733	0.085	2.279	0.023
	Total IQ	-0.083	0.023	-0.130	-3.639	0.000	-0.065	0.025	-0.088	-2.587	0.010	-0.080	0.023	-0.132	-3.552	0.000
	BIS (M)	2.619	0.510	0.214	5.133	0.000	4.136	0.563	0.295	7.347	0.000	0.607	0.506	0.052	1.201	0.230
Head and neck region	(Constant)	52.424	3.656		14.338	0.000	49.334	4.078		12.099	0.000	50.005	3.594		13.915	0.000
	Birth-assigned sex	-2.253	0.781	-0.108	-2.886	0.004	-4.697	0.871	-0.195	-5.394	0.000	1.387	0.767	0.070	1.808	0.071
	Age at intake	0.618	0.193	0.129	3.194	0.001	0.930	0.216	0.168	4.309	0.000	0.196	0.190	0.043	1.030	0.303
	Marital status	1.630	0.746	0.079	2.185	0.029	1.297	0.832	0.055	1.558	0.120	1.607	0.733	0.082	2.191	0.029
	Total IQ	-0.087	0.023	-0.136	-3.746	0.000	-0.070	0.026	-0.096	-2.727	0.007	-0.082	0.023	-0.134	-3.595	0.000
	BIS (M)	2.028	0.530	0.155	3.823	0.000	3.089	0.592	0.205	5.221	0.000	0.635	0.521	0.051	1.219	0.223
Muscularity and posture	(Constant)	53.028	3.637		14.578	0.000	50.275	4.074		12.335	0.000	50.190	3.578		14.029	0.000
	Birth-assigned sex	-2.649	0.756	-0.127	-3.505	0.000	-5.389	0.847	-0.223	-6.366	0.000	1.349	0.743	0.068	1.815	0.070
	Age at intake	0.509	0.198	0.106	2.578	0.010	0.869	0.221	0.157	3.928	0.000	0.061	0.194	0.013	0.313	0.755
	Marital status	1.487	0.744	0.072	1.999	0.046	1.115	0.833	0.047	1.339	0.181	1.526	0.732	0.078	2.086	0.037
	Total IQ	-0.086	0.023	-0.134	-3.735	0.000	-0.066	0.026	-0.090	-2.589	0.010	-0.084	0.023	-0.138	-3.709	0.000
	BIS (M)	2.593	0.570	0.186	4.549	0.000	3.321	0.638	0.207	5.202	0.000	1.418	0.561	0.107	2.530	0.012
Hip region	(Constant)	54.836	3.667		14.953	0.000	52.709	4.101		12.852	0.000	50.904	3.610		14.101	0.000
	Birth-assigned sex	-4.051	0.790	-0.193	-5.126	0.000	-7.263	0.884	-0.301	-8.217	0.000	0.741	0.778	0.037	0.952	0.341
	Age at intake	0.600	0.191	0.125	3.146	0.002	0.960	0.213	0.174	4.498	0.000	0.163	0.188	0.036	0.868	0.386
	Marital status	1.519	0.745	0.074	2.039	0.042	1.147	0.833	0.048	1.377	0.169	1.562	0.733	0.080	2.131	0.033
	Total IQ	-0.83	0.023	-0.131	-3.638	0.000	-0.064	0.026	-0.087	-2.505	0.012	-0.081	0.023	-0.134	-3.599	0.000
	BIS (M)	1.795	0.419	0.179	4.285	0.000	2.435	0.468	0.211	5.197	0.000	0.706	0.412	0.074	1.713	0.087
Chest region	(Constant)	52.710	3.694		14.270	0.000	49.925	4.140		12.058	0.000	50.117	3.611		13.877	0.000
	Birth-assigned sex	-3.560	0.797	-0.170	-4.468	0.000	-6.730	0.893	-0.279	-7.534	0.000	1.018	0.779	0.051	1.307	0.192
	Age at intake	0.771	0.190	0.161	4.052	0.000	1.151	0.213	0.208	5.395	0.000	0.257	0.186	0.056	1.380	0.168
	Marital status	1.527	0.753	0.074	2.027	0.043	1.123	0.844	0.047	1.330	0.184	1.583	0.737	0.081	2.149	0.032
	Total IQ	-0.077	0.023	-0.121	-3.347	0.001	-0.057	0.026	-0.078	-2.206	0.028	-0.079	0.023	-0.129	-3.474	0.001
	BIS (M)	1.035	0.439	0.098	2.355	0.019	1.627	0.493	0.134	3.304	0.001	0.247	0.430	0.024	0.574	0.566
Genital region	(Constant)	51.168	4.220		12.126	0.000	50.095	4.762		10.520	0.000	48.524	4.085		11.879	0.000
	Birth-assigned sex	-3.056	0.769	-0.146	-3.974	0.000	-5.927	0.868	-0.245	-6.830	0.000	1.199	0.744	0.061	1.611	0.108
	Age at intake	0.952	0.178	0.199	5.355	0.000	1.447	0.201	0.261	7.210	0.000	0.281	0.172	0.062	1.630	0.103
	Marital status	1.528	0.765	0.074	1.997	0.046	1.183	0.863	0.050	1.370	0.171	1.512	0.740	0.078	2.042	0.042
	Total IQ	-0.067	0.023	-0.105	-2.876	0.004	-0.049	0.026	-0.067	-1.869	0.062	-0.069	0.023	-0.115	-3.051	0.002
	BIS (M)	0.236	0.469	0.018	0.503	0.615	-0.087	0.529	-0.006	-0.164	0.870	0.235	0.454	0.019	0.518	0.604

BIS = Body Image Scale; YSR = Youth Self-Report, SE = standard error
